# Memorial Wreath for the Prosecution in the Graves Malpractice Suit

**Published:** 1886-12-20

**Authors:** Levi C. Lane

**Affiliations:** Professor of Surgery in Cooper Medical College, San Francisco


### MEMORIAL WREATH FOR THE PROSECUTION IN THE GRAVES MALPRACTICE SUIT.

BY LEVI C. LANE, M.D.,
Professor of Surgery in Cooper Medical College, San Francisco.

"*Quod medicinia non sanat, ferrum sanat; quad fernum non sanat, ignis sanat.*"—*Aphorism of Hippocrates.*

The medical profession of California have watched with special interest the case in which Dr. G. W. Graves, of Petaluma, was sued, over a year ago, for alleged malpractice. The cause of action was based on the charge of improper treatment of an injury to the ankle of a woman—one of those injuries where, despite the utmost care and skill on the part of the surgeon, the perfect use of the joint can never be regained. Though it was proven by written authority, as well as by prominent expert evidence, that the doctor had treated the case properly, and had obtained as good results as are gotten in such injury; yet facts, of which they became cognizant at the very commencement of the trial, have waged against the defendant during this long period a most merciless legal warfare, and one that has had but one parallel in the medical annals of this coast. And the outrage was further intensified by the fact that the plaintiff's family had received unremunerated services, during many years, from Dr. Graves.

Early in the case a compromise could have been effected, but the defendant, with more regard for his professional honor than for his purse, refused every overture of the kind; and, aided by the moral as well as the financial support of the profession, and inspired by the principle which guided our forefathers in their struggle, viz., *millions for defense, but not a cent for tribute*, he has made a resistance that deserves all praise, for a compromise, though it would have saved him money, would have been the signal for many similar suits against others.

The case has been tried three times, the first trial resulting in a verdict for eight thousand dollars against the defendant. This verdict, so at variance with what should have resulted from the evidence, awakened in the mind of almost every physician in this State a feeling of intense indignation and the determined resolve that the outrage should not be submitted to. At once, contributions were freely offered for the defense, amounting in the aggregate to nearly two thousand dollars. With this money additional legal counsel was employed, viz.: Hall McAllister, Esq., and Dr. E. R. Taylor, of this city; and, after no slight effort, the shameful verdict was set aside, and the case re-opened for trial.

At the second trial no decision was arrived at, the jury being divided; but at the third trial justice was triumphant, and victory crowned the side of right in a full acquittal of Dr. Graves.

This verdict, so gratifying to the defendant, is almost as much so to the general profession, of which several of the leading members, besides liberally contributing money, spent some days of precious time in attendance at court, to reach which, the most of them were compelled to travel long distances. But all will now feel more than repaid in the consciousness of having aided in rolling back the flood of injustice which was threatening to ingulf in ruin a professional brother. And besides this feeling of happiness, their united action has taught the lesson, that should a like instance recur, they will be quite as ready for concerted defense again; for, though the lance which they have lately so successfully wielded is laid in its rest yet all will see to it, that its point is kept sharp, and ready for action. All may be assured that this serpent, the foul offspring of communism, as often as it may be evoked from its slimy pool by legal incantations, will be returned again to its festering home without doing other violence than leaving its fangs in those who may conjure it forth. Whenever this Hercules, in the form of a malpractice suit, is sent forth in quest of golden apples, the profession are determined that he shall return, on this occasion, laden only with apples of Sodom, whose bitterness will leave an interminable writhing in the mouths of those who sent him on such a mission.

The fancy of Schiller has painted a strange scene, in which a young Moor is studying how he may shorten the life of his father he decided the surest plan would be to suddenly fill the old man's heart with intense happiness, and then, in a moment, to overwhelm him with grief and despair; in the course of this suit, this experiment, instead of in fancy, has been exhibited in reality. What wild joy the actors in this prosecution must have tasted when they obtained a verdict for eight thousand dollars; and with what heart-aches they must have been tortured when they saw the glitter of those ducats returning to the pocket of their rightful owner! Some curious psychologist would do well to chronicle how much their life-span has been shortened at its distal end by such experience.

Great praise is due to the attorneys who aided in the defense: Mr. McAllister, too long ago recognized as *facile princeps* at the bar of California, that any new salutations of praise can touch his ear, deserves thanks for snatching sometime from his overworked hours, and ably co-operating in the management which led the way to final victory in the case; and especially, is high credit due to Edward R. Taylor, who, though a graduate in medicine, has chosen the law as his profession; to his untiring work the final result is mainly due; for, besides bringing that special knowledge, which is so valuable in such a case, he threw his heart and soul into the matter, and worked with the zeal and enthusiasm of a personal friend of the medical profession.

The history of this case would be more satisfactory if it could close without reference to certain medical men who allied themselves with the plaintiff, and, as far as it was possible, aided in the prosecution. Some of these men are residents of San Francisco, and gave their evidence by deposition; but the two who were especially active in this work—unnatural as throwing stones at a mother—were present at the trial, and mingled in their evidence an amount of malevolence which has brought on them the universal contempt of the medical profession. Their position now finds a proper parallel in the case of the traitor Benedict Arnold, who, after the close of our Colonial war, being asked by some European for introductory letters to the New World, replied, that he was the only man in Europe who had no friends in America. As Ulysses, in his visit to Hades, being repelled by his old enemy Ajax, learned that the resentments of the dead are eternal, so these men will find that those of the living are no less so. Should they desire to return to the profession, whose altar they have sullied, ignominy, as a flaming sword, will forever prevent them. Such action, whithersoever it may turn, will find no rest; for should it think to find a screen for its offences in the flight of years, it will search in vain, since in the untrodden labyrinths of futurity, there will be found no hidden recess where the finger of infamy will not follow it; nay, more, death itself will give such action no refuge, since disgrace and dishonor will carve their initials upon its grâves-stone as an unfading epitaph.—*Pacific Medical Journal.*

